# Structure Dependent-Immunomodulation by Sugar Beet Arabinans via a SYK Tyrosine Kinase-Dependent Signaling Pathway

**DOI:** 10.3389/fimmu.2018.01972

**Published:** 2018-10-12

**Authors:** Marjolein Meijerink, Christiane Rösch, Nico Taverne, Koen Venema, Harry Gruppen, Henk A. Schols, Jerry M. Wells

**Affiliations:** ^1^Host Microbe Interactomics, Department of Animal Science, Wageningen University & Research, Wageningen, Netherlands; ^2^Top Institute Food and Nutrition, Wageningen, Netherlands; ^3^Laboratory of Food Chemistry, Wageningen University, Wageningen, Netherlands; ^4^Centre for Healthy Eating and Food Innovation, Maastricht University, Venlo, Netherlands; ^5^Beneficial Microbes Consultancy, Wageningen, Netherlands

**Keywords:** immunomodulation, dietary fiber, arabinans, pectin, structure-function relationship, C-type lectin receptors

## Abstract

There is much interest in the immunomodulatory properties of dietary fibers but their activity may be influenced by contamination with microbial-associated molecular patterns (MAMPs) such as lipopolysaccharide (LPS) and lipoteichoic acids, which are difficult to remove completely from biological samples. Bone marrow-derived dendritic cells (BMDCs) from TLR2x4 double-KO mice were shown to be a reliable approach to analyse the immunomodulatory properties of a diverse range of dietary fibers, by avoiding immune cell activation due to contaminating MAMPs. Several of the 44 tested dietary fiber preparations induced cytokine responses in BMDCs from TLR2x4 double-KO mice. The particulate fractions of linear arabinan (LA) and branched arabinan (BA) from sugar beet pectin were shown to be strongly immune stimulatory with LA being more immune stimulatory than BA. Enzymatic debranching of BA increased its immune stimulatory activity, possibly due to increased particle formation by the alignment of debranched linear arabinan. Mechanistic studies showed that the immunostimulatory activity of LA and BA was independent of the Dectin-1 recognition but Syk kinase-dependent.

## Introduction

Dietary fiber has been defined by the European Commission as “carbohydrate polymers possessing three or more monomeric units, which are neither digested nor absorbed in the human small intestine” (1). This definition includes carbohydrate polymers occurring naturally in foods as well as those obtained by physico-chemical treatments of raw food materials or via synthetic approaches. Categorization of diverse fiber structures is typically based on chemical properties such as monosaccharide constitution or physical properties such as solubility (2). The fermentation of different fibers by intestinal microbiota is of growing interest as it generates short chain fatty acids (SCFA). These metabolites have profound effects on host physiology, ranging from energy metabolism (3–7), enteroendocrine function (8, 9), barrier protection (10), and the induction of regulatory T cells in the colon (11–15).

Several studies have shown a direct immunostimulatory effect of dietary β1,3-linked glucans on immune cells. These fibers are found in the cell walls of mushrooms and other fungi and trigger a variety of immune cell responses including maturation of myeloid cells, the respiratory burst in innate phagocytes, and production of cytokines (16). These responses are mediated through binding of the β1,3-linked glucans to dectin-1, a member of the C-type lectin receptor (CLR) family (17). Dectin-1 is utilizing a tyrosine kinase (SYK) as a primary signal transduction molecule to induce inflammatory responses (18). Recruitment of SYK is thought to be dependent on the bridging of two phosphorylated immunoreceptor tyrosine-based activation (ITAM) domains by the dimerization of dectin-1 (18). Dectin-1 and other CLRs are expressed on dendritic cells and macrophages, subsets of which are associated with intestinal epithelial cells and can sample antigens directly through the epithelium. Recently dectin-1 was also shown to be expressed in primary colonic enterocytes and induce chemokine secretion upon ligand binding to β1,3-linked glucans (17). Evidence that dectin-1 signaling is important in the gut comes from the finding that a loss of function polymorphism in dectin-1 is associated with more severe forms of ulcerative colitis (19). Little is known about the expression and function of other CLRs in the intestinal epithelium, such as dectin-2 and macrophage inducible Ca^2+^-dependent (C-type) lectin (Mincle).

A large body of literature describes immune stimulation with different types of dietary polysaccharides including pectins, heteroglycans, glucomannans, fucoidans, galactomannans, arabinogalactans (20, 21), but with exception of β1,3-linked glucans the molecular basis of the signaling interactions are poorly understood. One of the reasons has been the presence of contaminating microbial-associated molecular patterns (MAMPs) such as lipopolysaccharide (LPS) and lipoteichoic acids in dietary fiber sources (22–24). These MAMPs are difficult to remove completely in biological samples making it impossible to definitively determine whether the carbohydrates or the contaminating MAMPs induce the cellular responses.

The aim of this study was to explore the immune stimulatory activity of a panel of 44 dietary fibers and select an active fiber to determine the signaling mechanism and structural component linked to the immune stimulatory activity. To avoid measuring responses to LPS, LTA and lipoprotein components that might contaminate the fibers we used bone-marrow derived dendritic cells (BMDCs) from mice lacking TLR2 and TLR4. Based on the first screen, we chose to investigate the relationship between immune stimulatory activity and the chemical properties of different carbohydrate structures derived from sugar beet pectin (SBP). Mechanistic studies were then performed to determine whether dectin-1 or other the SYK-dependent carbohydrate receptors were involved in the immunostimulatory effects.

## Materials and methods

### Substrates

The collection of 44 fiber substrates, obtained from different commercial sources and laboratories, represent a diverse range of plant derived dietary fiber. These fibers have been categorized, based on their monosaccharide constitution, into 7 groups and the manufacturers information is provided in Table 1.

**Table 1:** Monosaccharide composition of the analyzed dietary fiber.

	**Sample name (according to supplier)**	**Supplier**	**mol %**							**Total w/w%**	**Protein w/w%**
			**Rha**	**Ara**	**Xyl**	**Man**	**Gal**	**Glc**	**Uronic acid**		
Starchy material	Nutriose FM 06, RS 4	Ingredion (Westchester, IL, USA)	0	0	0	0	0	99	1	86	0
	Nutriose FB 06, RS 4	Ingredion (Westchester, IL, USA)	0	0	0	0	0	99	1	83	0
	Soluble corn fiber, RS 4	Tate & Lyle (London, United Kingdom)	0	0	0	0	0	99	1	90	0
	Sta-Lite Polydextrose, RS 4	Tate & Lyle (London, United Kingdom)	0	0	0	0	0	97	1	90	0
	Fibersol-2, RS 4	ADM/Matsutani LLC (Hyogo, Japan)	0	0	0	0	0	99	1	85	1
	Resistant wheat dextrin, RS 4	Roquette (Lestrem, France)	0	0	0	0	0	99	1	87	0
	Maize, RS 3	Nutricia Research (Utrecht, The Netherlands)	0	0	0	0	0	99	1	87	3
	Novelose 330, RS 3	Ingredion (Westchester, IL, USA)	0	0	0	0	0	99	1	82	2
	High maiz 260, RS 2	Ingredion (Westchester, IL, USA)	0	0	0	0	0	99	1	85	2
Cellulosic fibers	Cellulose	Nutricia Research (Utrecht, The Netherlands)	0	0	19	0	0	80	1	66	0
	Wheat cellulose	J. Rettenmaier & Söhne (Rosenberg, Germany)	0	2	27	0	0	70	2	81	0
	Oat bran	Megro GmbH & Co KG (Eppelborn, Germany)	0	2	3	0	0	91	3	59	19
	Carboxymethyl cellulose	Sigma-Aldrich (St. Louis, MO, USA)	0	0	3	0	0	94	3	n.a.	0
	Microcrystalline cellulose	Brunschwig Chemie (Amsterdam, The Netherlands)	0	0	2	2	0	95	1	53	0
Extracted pectic fibers	Lemon pectin DM67	CP Kelco (Copenhagen, Denmark)	1	2	0	0	4	1	92	86	2
	Lemon pectin DM30	CP Kelco (Copenhagen, Denmark)	0	0	0	0	3	0	96	82	3
	Apple pectin DM63	Herbstreith & Fox KG (Neuenbürg, Germany)	1	3	2	0	6	2	87	72	13
	Apple pectin DM41	Herbstreith & Fox KG (Neuenbürg, Germany)	1	1	1	0	4	2	92	83	2
	Sugar beet pectin (SBP)	DuPont (Brabrand, Denmark)	2	6	0	0	12	2	78	73	5
	Linear arabinan (LA)	British sugar (Peterborough, United Kingdom)	3	60	0	0	17	6	13	69	3
	Branched arabinan (BA)	British sugar (Peterborough, United Kingdom)	1	74	0	1	13	3	9	67	2
	Soy pectin	Fuji Oil Co. Ltd. (Osaka, Japan)	2	27	7	0	42	3	20	68	5
	Pectin hydrolysed	Nutricia Research (Utrecht, The Netherlands)	1	2	0	0	4	1	92	93	3
	Potato galactan	Megazyme (Bray, Ireland)	2	4	0	0	73	1	20	66	2
Crude fibers	Wheat bran	The King Milling Company (Lowell, MA, USA)	0	18	32	1	2	42	4	48	14
	Barley bran	Cargill (Vilvoorde, Belgium)	0	7	11	1	0	79	2	58	18
	Rye bran	CSM (Bremen, Germany)	0	12	33	2	2	47	4	55	16
	Oat cellulose	White Star GmbH (Thannhausen, Germany)	0	4	44	0	0	50	3	75	0
	Soy bran	Caj. Strobl-Naturmühle GesmbH (Linz-Ebelsberg, Austria)	1	9	11	9	6	43	21	48	7
	Soy fiber	Fuji Oil Co. (Osaka, Japan)	1	20	8	2	36	18	16	68	10
	Apple fiber	GoodMills Innovation GmbH (Hamburg, Germany)	1	11	5	8	6	45	24	55	7
	Sugar beet cellulose	Nordic sugar A/S (Copenhagen, Denmark)	2	28	3	2	7	26	33	63	9
Hemicelluloses	Wheat arabinoxylan	Megazyme (Bray, Ireland)	0	35	67	0	0	0	0	82	1
	Rye arabinoxylan	Megazyme (Bray, Ireland)	0	31	67	0	1	0	1	74	1
	Arabinoxylan	Kelloggs (Battle Creek, MI, USA)	0	14	69	0	0	15	1	85	0
	Oat β-glucan	Megazyme (Bray, Ireland)	0	0	0	0	0	99	1	100	1
	Barley β-glucan	Megazyme (Bray, Ireland)	0	0	0	0	0	99	1	99	0
Gums	Gum guar	C.E. Roeper GmbH (Hamburg, Germany)	0	3	1	57	34	4	2	73	6
	Locust bean gum	CP Kelco (Copenhagen, Denmark)	0	0	0	64	16	17	2	80	0
	Gum arabic	Nutricia Research (Utrecht, The Netherlands)	4	43	0	0	39	0	14	73	1
Prebiotics	GOS Vivinal	Friesland Campina (Wageningen, The Netherlands)	0	0	0	0	48	51	1	51	1
	FOS Chicory	Nutricia Research (Utrecht, The Netherlands)	F:G = 22							91	0
	Inulin chicory	Nutricia Research (Utrecht, The Netherlands)	F:G = 13							95	0
	Inulin chicory high DP	Nutricia Research (Utrecht, The Netherlands)	F:G = 45							88	1

*RS, resistant starch; Rha, Rhamnose; Fuc, Fucose; Ara, Arabinose; Xyl, Xylose; Man, Mannose; Gal, Galactose; Glc, Glucose; Fructose, G, Glucose; DP, degree of polymerization*.

### TLR assays

TLR signaling capacities of the fibers were determined using a reporter assay with human embryonic kidney (HEK) 293 cells (Invivogen, Toulouse, France). These cells expressed: human TLR2 and TLR6 heterodimers, that recognize lipoteichoic acid (LTA) and lipoprotein lipid anchors of Gram-positive bacteria (25); human TLR2; human TLR1/2; human TLR4, that recognizes LPS and TLR5, that recognizes flagellin. The TLR signaling assays were performed essentially as previously described (26). Briefly, HEK293 cells were transfected with one of the mentioned human TLR(s) and pNiFty-luc, a NF-κB luciferase reporter construct (Invivogen, Toulouse, France, catalog numbers 293-htlr2; 293-htlr4a; 293-htlr5; 293-htlr2/6; 293-mtlr1/2; pnifty-luc). Additionally, HEK293 cells were transformed with only the pNiFty-luc (26). The cells were plated at a concentration of 6 × 10^4^ cells per well in DMEM medium (Invitrogen, Breda, The Netherlands) and incubated overnight at 37°C. Cells were then stimulated with the different fibers (400 μg/mL), or positive controls in different doses: LPS (1–750 ng/ml), LTA (0.312–20μg/mL), flagellin (1.3–1,000 ng/mL), Pam3-CSK_4_, a tripalmitoylated lipohexapeptide analog of the immunologically active N-terminal portion of bacterial lipoprotein (64–1,000 ng/mL) and Pam2-CSK_4_, a dipalmitoylated lipohexapeptide analog of the immunologically active N-terminal portion of bacterial lipoprotein (40 ng/mL) (all supplied by Invivogen, Toulouse, France, catalog numbers: tlrl-pms; tlrl-pm2s-1; tlrl-pslta; tlrl-epstfla) or with medium alone (negative control). The assays were incubated at 37°C for 6 h under a 5% CO_2_ atmosphere. Subsequently, the medium was replaced with Bright glow (Promega, Leiden, The Netherlands), and the plates shaken for 5 min before measuring the luminescence in a Spectramax M5 (Molecular Devices, Sunnyvale, United States).

### Generation of mouse bone-marrow derived dendritic cells (BMDCs)

To obtain BMDC cells, 6–10 weeks old TLR2x4 double-KO C57bl/6 mice (27) were euthanized, femurs were isolated, washed and gently crushed in 10 mL of RPMI-1640 medium (without HEPES) supplemented with 100 units/mL penicillin G (Invitrogen) and 100 μg/mL streptomycin (Thermo Fisher catalog number: 15140122). Cells were filtered using a 40 μm Steriflip® Filter Unit (Merkmillipore, catalog number SCNY0040) and around 2-4 × 10^4^ cells per well were seeded in a 96-well flat bottom plate in complete media (RPMI-1640 medium, containing: 10% heat-inactivated fetal calf serum Gibco catalog number 26140-079; 100 units/mL, penicillin G, 100 μg/mL streptomycin, supplied as above; 20 ng/mL of recombinant mouse granulocyte-macrophage colony-stimulating-factor, R&D systems catalog number 415-ML-010 and 0.05 mM of beta-mercaptoethanol, Thermo Fischer catalog number 31350010). Cells were incubated at 37°C in CO_2_ atmosphere and medium was changed every 3 days. At day six BMDCs express low activation markers (not shown) and cytokine production is below level of reliable detection in the cytometric multiplex bead assay. However, stimulation with positive controls (e.g., zymosan) induces production of high amounts of cytokine after 24 h (Supplementary Figure 2). On day six, BMDCs were stimulated with 400 μg/mL fiber (or different fiber concentrations in some assays), TLR ligands as mentioned above or depleted zymosan (20 μg/mL), lacking TLR signaling activity. Where indicated in the figures, 10 μM SYK kinase inhibitor piceatannol (Sigma-Aldrich, Netherland, catalog number P0453-5MG) or 10 μg/mL blocking IgG antibody to anti-dectin-1 (InvivoGen, Toulouse France, catalog number mabg-hdect) or 10 μg/mL IgG1 isotype antibody control (InvivoGen, Toulouse France, catalog number mabg1-ctrlm) were also added.

### Cytokine assays

Supernatants from the BMDC stimulation assays were collected after stimulation for 24 h. A cytometric bead-based BD mouse inflammation kit (BD Biosciences, Breda, the Netherlands, Fisher Scientific, catalog number 552364) was used to measure the cytokines IL-12p70 (from here on referred to as IL-12), IL-10, interferon-γ (IFNγ) (from here on referred to as IFN), TNF, IL-6 and the chemokine MCP-1. This kit was used for multiplex measurements of cytokines in cell supernatants by flow cytometry according to the manufacturer's protocol (BD Biosciences, Breda, the Netherlands) (28). The sensitivity-limits of detection, as indicated by the supplier, were as follows: IL-12 10.7 pg/mL, IL-10 17.5 pg/mL, IFN 2.5 pg/mL, MCP-I 52.7 pg/mL, TNF 7.3 pg/mL and IL-6 5 pg/mL. IFN levels were all below detection limit and are therefore not shown. The flow cytometry data were analyzed using the BD FCAP software.

### Characterization of dietary fibers

#### Constituent monosaccharide composition

The constituent monosaccharide composition was determined using a pre-hydrolysis step with 72 % (w/w) sulphuric acid at 30°C for 1 h, followed by hydrolysis with 1 M sulphuric acid at 100°C for 3 h. The monosaccharides released were derivatized to alditol acetates and analyzed by gas chromatography using inositol as an internal standard (29). Uronic acid (UA) in the samples was determined by using the colorimetric m-hydroxydiphenyl assay (30) automated on an autoanalyser (Skalar, Breda, The Netherlands) as previously described (31).

#### High performance size exclusion chromatography (HPSEC)

The arabinan solutions (5 mg/mL water, RT) were centrifuged (10 min, RT, 18,000 × g) and the supernatant was analyzed for molecular weight distribution with high performance size exclusion chromatography (HPSEC) on an Ultimate 3000 HPLC (Dionex, Sunnyvale, CA, USA) equipped with a Shodex RI-101 refractive index detector (Showa Denko K.K., Tokyo, Japan). Three TSK-Gel columns connected in series (4000-3000-2500 SuperAW; 150 × 6 mm) were used for the analysis. These columns were preceded by a TSK Super AW-L guard column (35 × 4.6 mm). All columns were from Tosoh Bioscience (Tokyo, Japan) and covered a molecular weight range from 0 to 250 kDa. Twenty μL sample were injected and eluted with 0.2 M NaNO_3_, at 40°C with a flow rate of 0.6 mL/min. Pullulan molecular-mass standards (Polymer Laboratories, Palo Alto, CA, USA) were used for calibration.

#### Particle size distribution

The particle size of linear arabinan (LA), branched arabinan (BA) and soy-bean pectin (SBP) was determined using laser light diffraction (Mastersizer 2000, Malvern Instruments Ltd., Malvern, United Kingdom) equipped with a Hydro SM sample dispersion unit. The suspensions in water were analyzed in triplicate and averaged. To derive the particle size, the Fraunhofer model with an absorption of 0.1 for the particles and a refractive index of 1.33 for water was used.

#### Chemical and physical treatments of fibers

Several methods were used to modify or separate the fibers into different fractions and to investigate the capacity of these fractions to activate BMDCs and induce cytokine production.

#### Separation by water extraction

LA, BA and SBP were suspended in water (10 mg/mL), solubilized for 2 h, then centrifuged (10 min, RT, 18,000 × g) and both supernatant and particulate fraction were freeze-dried. The fractions were analyzed for their monosaccharide constituent composition and immune-activity on BMDCs as described. In this study the supernatant fraction is referred to as “soluble” and the particulate fraction as “particulate.”

#### Ethanol extraction

In order to test if proteins or other a-polar non-carbohydrates, present in the substrate, were inducing cytokines in BMDCs, the substrates were suspended in 70% (v/v) ethanol at a concentration of 10 mg/mL at RT, then centrifuged (10 min, RT, 18,000 × g) and separated by decanting. After evaporating the ethanol, the soluble fraction and the pellet were freeze dried. The fractions were analyzed for their immune-activity and the monosaccharide constitution was determined.

#### Size separation by sieving

LA, BA, and SBP were separated by sieving (106 μm sieve; Retsch, Haan, Germany) into fractions smaller and larger than 106 μm, and designated with the prefix “small” or “large.” The sieve shaker type AS200 digit (Retsch) was set to an amplitude of 50 for 1 min.

#### Enzyme treatment

Arabinofuranosidase was used to remove arabinose-side chains from the (highly-branched) BA to linearize it and generate a structure similar to LA. The BA (5 mg/mL) in 50 mM NaOAc buffer pH 5 was incubated with 30 μL arabinofuranosidase [*Chrysosporium lucknowense* (29), 0.95 U/mg] at 40°C at 600 rpm for 0.5, 2, 4, 7, 24, and 48 h. The reaction was stopped by heating the samples for 10 min at 100°C in a water bath. Blanks prepared in buffer, containing only the enzyme and arabinans were incubated in the same way as the fiber samples. The incubations were performed in duplicate and the enzyme digesta were analyzed with HPSEC. The immune-activity was analyzed after freeze-drying of the sample.

#### Statistics

All statistical tests were performed using GraphPad Prism 5.0 software (GraphPad, San Diego, California). Data shown are the means and the standard errors of the means (SEM). Data were tested for normality with the D'Agostino and Pearson normality test. Statistical analysis of normally distributed data was performed with the 2 tailed unpaired *T*-test. Data that did not show normal distribution were analyzed with the Welch's correction to determine equal variances between the groups. When the variances were unequal between the groups, the data was analyzed using the unpaired *T*-test with Welch's correction. Differences were considered statistically significant when the *P*-value was ≤ 0.05.

## Results

### Categories of fibers included in the screen

The collection of 44 dietary fibers were categorized due to their chemical characteristics into 7 classes: starchy material, cellulosic fibers, pectic fibers, brans, hemicelluloses, gums and prebiotics (Table 1). The fibers of each category have all a common structural characteristic, but within each category variations in monosaccharide and linkage composition, solubility and particle size are possible. Such differences in physicochemical characteristics might lead to differences in immunomodulatory activity as discussed below.

The fibers in the group of starchy materials are resistant starches (RS) of different type (RS type 2, 3, and 4). The RS 3 samples consist of higher portions of digestible starch and are partly insoluble. All starchy materials are mainly α-linked glucose units, but connected through different glycosidic linkages, which results in different conformations. RS 4 samples are soluble and consist of diverse types of chemically modified linkages.

The fibers of the category cellulosic fibers consist of β-1,4 linked glucose moieties (32). The cellulosic substrates of this study are commercial products with cellulose as the main component. However, depending on the manufacturing process, they still contain hemicelluloses (arabinoxylan).

The pectins, which are a heterogeneous and more complex class of dietary fiber all possess a homogalacturonan backbone, which is differently decorated with methyl esters and/or acetyl groups. The rhamnogalacturonan I backbone has arabinan and/or galactan sidechains attached. Modification of the pectin backbone depends on the plant source of the pectin and influences solubility (33, 34).

Hemicelluloses are represented by β-glucans and arabinoxylans from wheat, rye and oat. Arabinoxylans consist of different ratios of arabinose and xylose. β-1,4 Linked xyloses form a backbone, which is mono- or di-substituted by arabinose in O-2 or O-3 position (35). The hemicellulose of cereal β-glucan consists of only β1,3-1,4-linked glucose moieties, which vary in their DP3/DP4 ratio (36, 37).

The substrates presented in the prebiotic category are higher oligomers consisting of glucose linked with different amounts of fructose or galactose, for fructooligosaccharides (FOS) or galactooligosaccharides (GOS) (36–38), respectively. The sample inulin chicory high DP consists of only high DP fructans (> DP 10) up to DP 30. All three fructans consist of different fructose/glucose ratios as indicated in Table 1.

The crude fibers are milling by-products from flour production, soy bean flour from the oil production and remaining apple pomace from juice production. Crude fibers are typically characterized as complex mixture of different fibers such as cellulose, β-glucan, and arabinoxylan in different ratios and of different structures, depending on the source (7). Additionally, the crude fiber samples contained relatively high amounts of proteins (7–19 w/w%) in comparison to the rest of the fiber samples (<6%) (data not shown).

The 44 dietary fibers were categorized according to similarity of common chemical-structural characteristics, but within each category every fiber has a unique chemical structure, which leads to unique substrate properties. The monosaccharide constitution of the different plant-derived dietary fibers was determined (Table 1).

### TLR signaling by fiber samples

In a preliminary screen, several of the 44 fibers described above activated human peripheral blood mononuclear cells (PBMCs) suggesting they might have immune stimulatory activities (data not shown). To be able to distinguish between immune stimulatory activities of the fibers and that of contaminating MAMPs, we screened each fiber for their capacity to induce TLR signaling, as this would indicate potential contamination with MAMPs. To detect specific TLR signaling activity we used embryonic HEK293 reporter cells expressing different human TLR receptors and carrying the reporter plasmid pNiFty, which expresses luciferase in response to NF-κB activation. A control cell reporter harboring only pNiFty, did not respond to any TLR agonists but as expected expressed luciferase in response to TNF-α (5 ng/ml) (Supplementary Figure 1). While none of the TLR2 agonists tested (i.e., Pam2-CSK_4_, lipoteichoic acid and Pam3-CSK_4_) activated NF-κB in the TLR4 reporter cell line, LPS, a TLR4 agonist, induced NF-κB activation in the TLR2 reporter cell line. This was likely due to cross-recognition of LPS by via the lipid-binding pocket in TLR2. In the TLR5 reporter cell line only flagellin activated NF-κB whereas flagellin weakly activated NF-κB in the TLR2 and TLR4 reporter cells at the highest concentrations, possibly due to contamination with small amounts of other bacterial MAMPs (Supplementary Figure 1).

**Figure 1:**
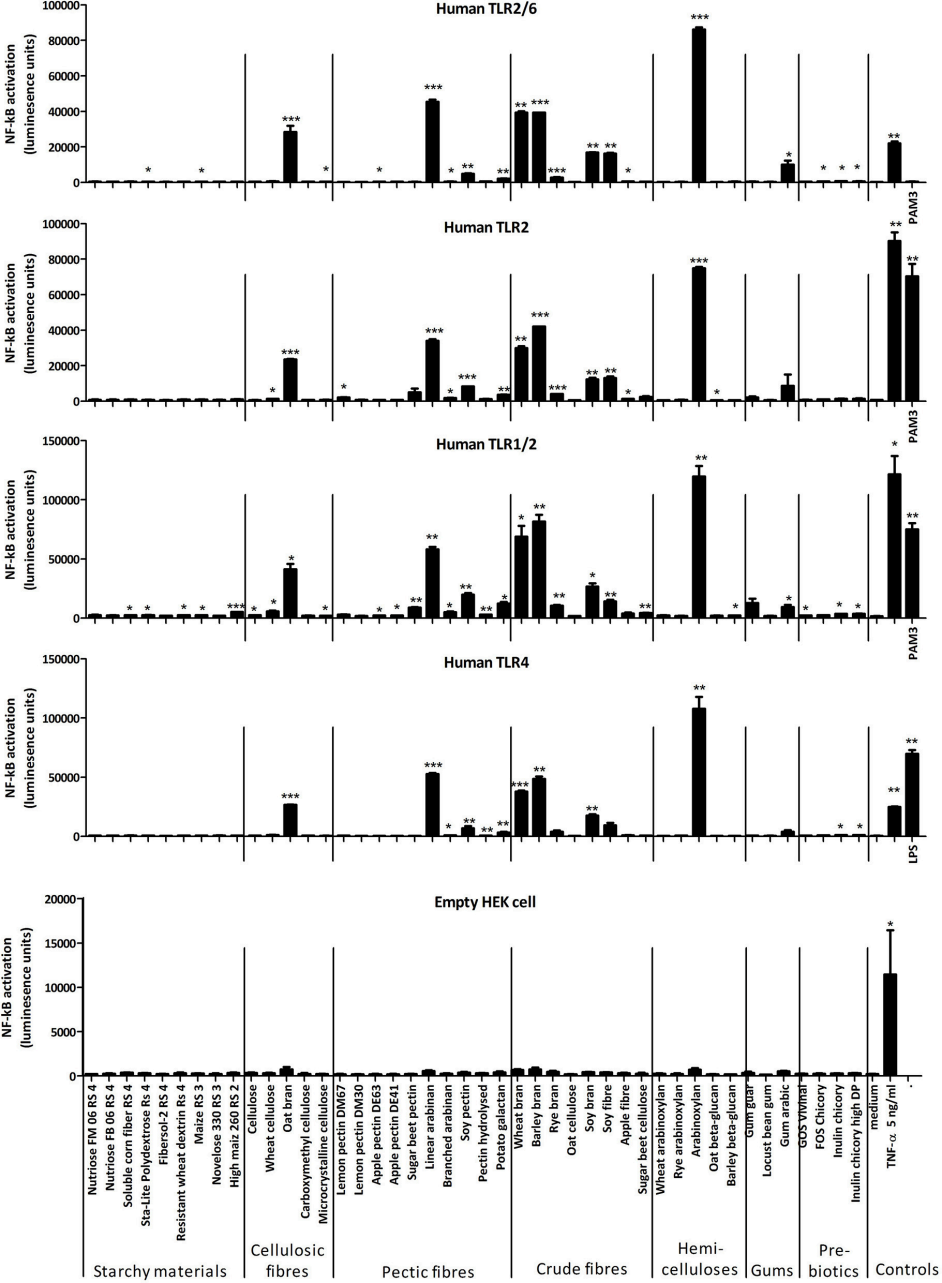
Mean luciferase activity of HEK293 TLR reporter cell lines incubated with different fibers (at approx. 400 μg/mL) and positive controls [TNF (5 ng/mL), Pam3-CSK_4_ (200 ng/mL), and LPS (250 ng/mL)]. Error bars represent the standard error of the mean values. Asterisks represent classes of statistically significant different responses compared to the medium control (*P < 0.05, **P < 0.01, and ***P < 0.0001).

Some of the immune stimulatory fibers, notably arabinoxylan, most brans, linear arabinan and some others of the pectic fiber category strongly activated NF-κB in both the TLR2 and TLR4 reporter cells, suggesting they were contaminated with bacterial MAMPs (Figure 1). Indeed, measurements of LPS revealed that it was present in the above-mentioned fiber substrates (data not shown). None of the fibers significantly activated NF-κB in HEK293 cells carrying only pNiFty and not expressing various types of pattern-recognition receptors (PRRs) that recognize carbohydrate structures, for example, C-type lectin receptors (CLRs) such as dectin-1.

### CLR but not TLR agonists induce cytokine secretion in BMDCs from TLR2x4 double-KO mice

To investigate whether fibers inducing TLR2 and TLR4 signaling would stimulate immune responses via a TLR-independent mechanism they were incubated with bone marrow derived dendritic cells (BMDCs) from TLR2x4-double KO mice. Dendritic cells (DCs) were chosen because they are known to express several receptors of the C-type lectin family that might bind to carbohydrate structures in fibers (39). To validate the suitability of these cells, BMDCs from TLR2x4 double-KO mice were stimulated with a wide range of concentrations of different purified agonists (LPS, LTA, flagellin, Pam_3−_CSK_4_) (Supplementary Figure 2). None of the TLR2 and 4 agonists tested elicited significant amounts of cytokines, while they induced strong immune responses in BMDCs from wild-type mice (data not shown). Flagellin did not elicit cytokine responses in BMDCs from knockout or wild type mice, but a similar finding was previously described for flagellin using BMDCs (40). Zymosan (20 μg/mL), depleted of TLR activity, induced cytokine responses in BMDCs (Supplementary Figure 2). These results showed that BMDCs from TLR2x4 double-KO mice are not activated by TLR2, 4 or 5 agonists but can be activated by the interaction of zymosan with the C-type lectin receptor Dectin-1.

### Several fibers induce cytokine secretion in BMDCs from TLR2x4 double-KO mice

**Figure 2:**
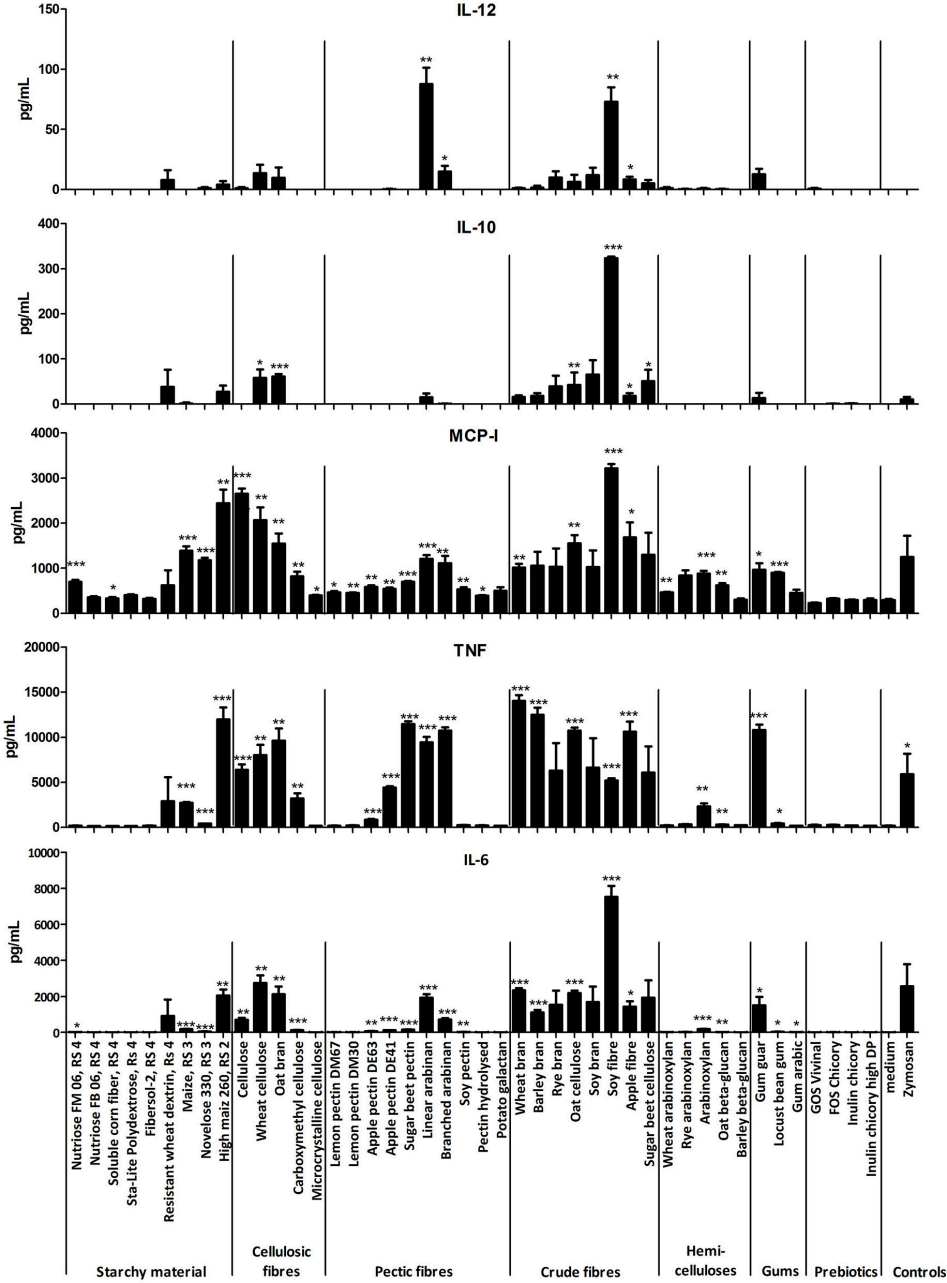
Mean amounts of cytokine or MCP-1 chemokine produced after incubation of BMDC from TLR2x4 double-KO mice with different fibers (400 μg/mL) or depleted zymosan (20 μg/mL) as a positive control. Error bars represent the standard error of the mean values. Asterisks represent classes of statistically significant different responses compared to the medium control (*P < 0.05, **P < 0.01, and ***P < 0.0001).

Several fibers including many of the category bran, cellulosic fiber and pectic fibers (SBP, LA, and BA) and some resistant starches (RS type 3), induced significant secretion of cytokines in BMDCs from TLR2x4 double-KO mice compared to non-stimulated control cells (Figure 2). As these BMDCs did not respond to high concentrations of bacterial MAMPs (Supplementary Figure 2), we concluded that the fibers were inducing cytokine secretion through interactions with different pattern recognition receptors, for example CLRs. Further evidence for this hypothesis comes from the finding that some fibers, such as cellulosic fibers, did not possess any TLR signaling activity in TLR reporter cell lines, but were signaling in the BMDC assays (Figures 1, 2). The responses to fibers were not due to cell toxicity as we determined the proportion of live cells using an Annexin V/propidium iodide staining in BMDCs stimulated with different fibers, depleted zymosan and in unstimulated cells and the fiber stimulations did not induce cell death (data not shown). Some dietary fibers induced strikingly different amounts of cytokines (Figure 2), even though they were of the same class e.g., brans and some pectic fibers, suggesting that their immunomodulatory properties are influenced by the different physicochemical properties of each fiber.

The immune stimulatory effects of the fibers were further investigated by washing the fiber suspension with ethanol (EtOH) to remove apolar non-carbohydrate compounds that might have an immune-stimulating activity. The suspension in ethanol was separated in soluble and particulate fractions, freeze-dried and tested at the same concentration (400 μg/mL) for their capacity to stimulate cytokine secretion by BMDCs from TLR2x4 double-KO mice. Most of the ethanol fractions did not induce cytokine secretion, however those that did were from small oligosaccharides or other (polar) compounds, which had been solubilized in the EtOH (data not shown). Overall, immunomodulatory properties are specific for each fiber and a general conclusion about the immunomodulatory properties cannot be based simply on the fiber category.

### Sugar beet pectin, linear arabinan and branched arabinan differentially induce cytokine secretion in BMDCs

**Figure 3:**
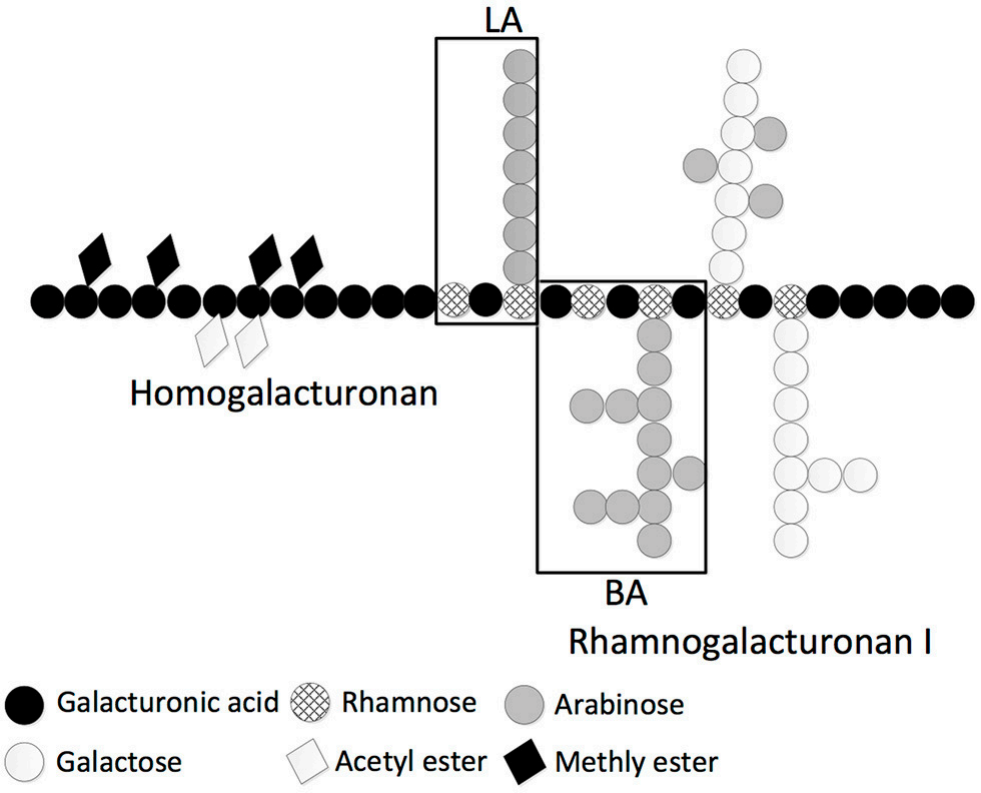
Schematic representation of sugar beet pectin substructures homogalacturonan, rhamnogalacturonan I, LA and BA on monosaccharide constitution level [Adapted from Pedrolli et al. (43)].

We decided to focus further studies on the physicochemical and immune stimulatory properties of two carbohydrate polymers derived from sugar beet pectin (41, 42). Commercial SBP is an acid extract from the sugar beet pulp and only short galactan and arabinan side chains are present. Branched arabinan (BA) is chemically derived from native SBP and represents mainly the sidechains of the rhamnogalacturonan I region of the native pectin. Linear arabinan (LA) is derived from BA by enzymatic removal of the arabinan side chains (Figure 3). LA, BA, and SBP consist of 19, 2, and 63 w/w% particulate material, respectively (RT, 10 mg/mL).

**Figure 4:**
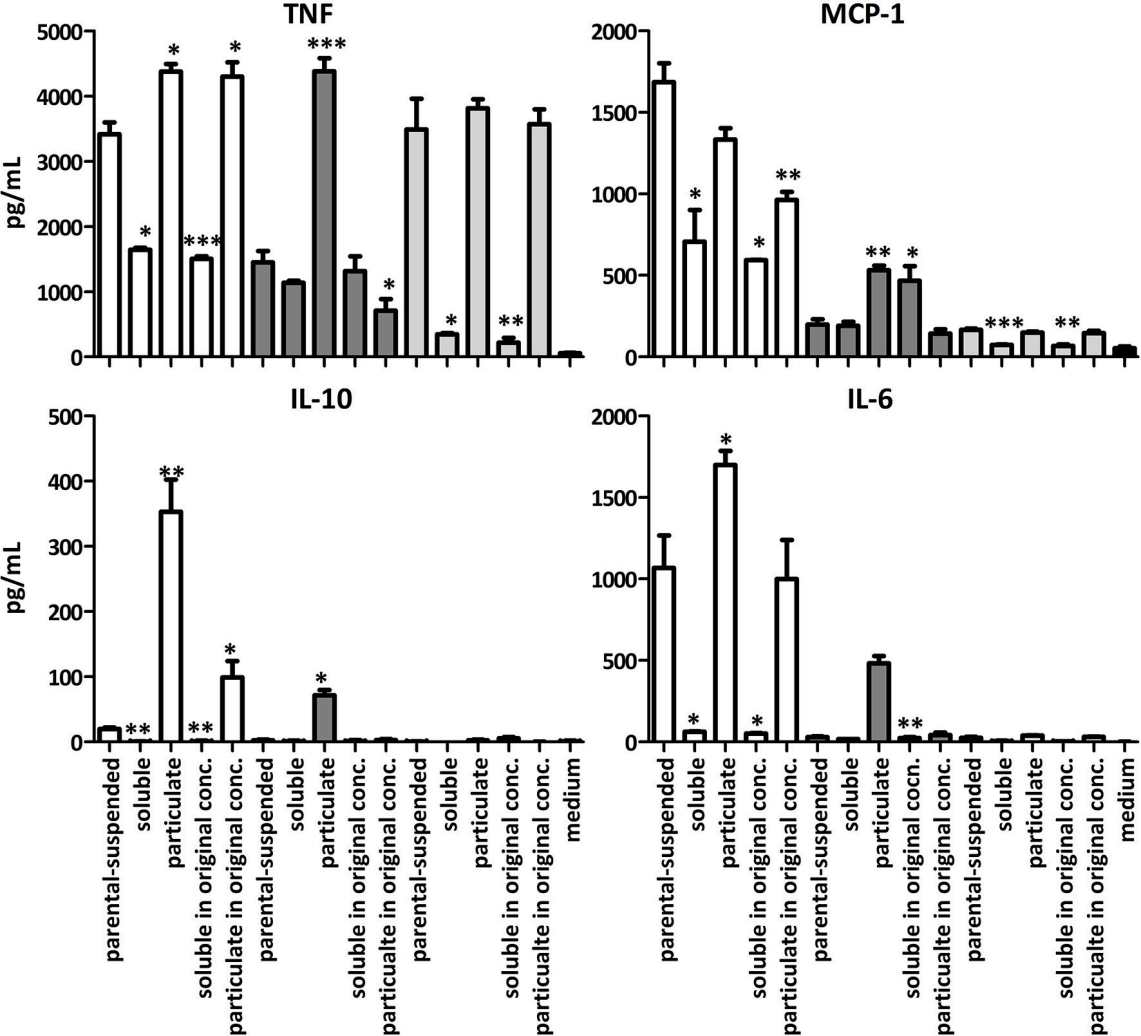
Mean amounts of cytokine or MCP-1 chemokine produced after incubation of BMDC from TLR2x4 double-KO mice fiber suspensions of the fractionated soluble and particulate components. LA (white), BA (dark gray) and SBP (light gray). Error bars represent the standard error of the mean values. Asterisks represent classes of statistically significant different responses compared to the suspension of the fiber (*P < 0.05, **P < 0.01, and ***P < 0.0001).

To identify the bioactive compound, the suspended, soluble and particulate fractions of LA, BA and SBP were incubated with BMDCs from TLR2x4 double-KO mice. The particulate LA and BA fractions induced significant amounts of TNF, IL-6, IL-10, and MCP-1 compared to the suspended and soluble samples (Figure 4). However, the MCP-1, IL-10, and IL-6 responses to particulate BA were lower than that to particulate LA at the same concentration. SBP samples tested induced significant amounts TNF and MCP-1, but not IL-10 or IL-6 (Figure 4).

When the soluble and particulate fractions of LA were incubated with BMDCs at the concentration found in the original sample or parenteral suspension (i.e., 81 w/w% soluble and 19 w/w% particulate), it was apparent that the soluble LA antagonized the immune stimulatory activity of particulate LA molecules. This conclusion is based on the fact that the particulate LA induces larger amounts of cytokines in BMDCs than the suspension of the LA before fractionation even though amounts to only 19 w/w% of the total mass. Particulate BA, which makes up only 2 w/w% of the whole substrate induces about 50% of the amounts of cytokines induced by the suspension of BA before fractionation (Figure 4) showing that the particulate BA has most immune stimulatory activity. To further explore the reasons for the immune-modulating differences between these three fibers we investigated their chemical and physical characteristics.

### Particle size distribution of the arabinans

**Figure 5:**
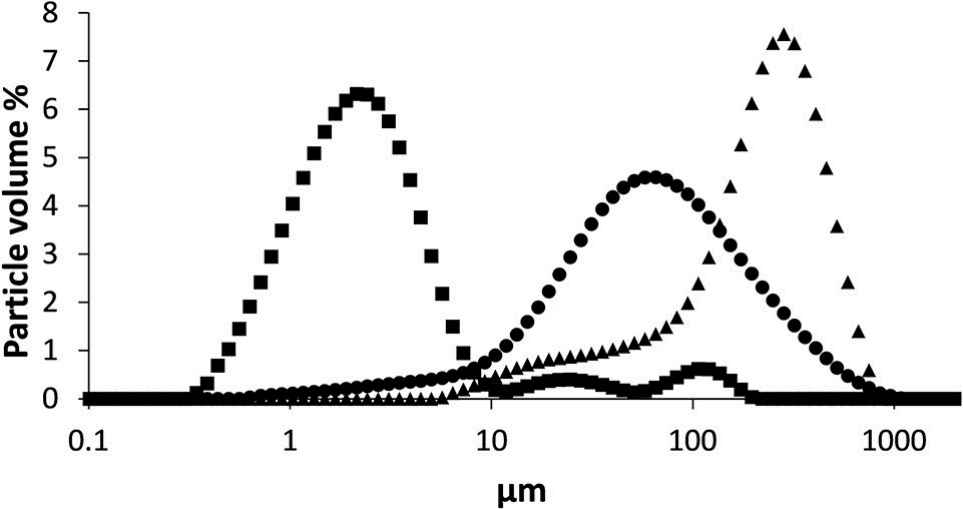
Particle size (μm) distribution in volume % of suspended BA (■), LA (●), and SBP (▲) measured by laser light scattering.

As the particulate fractions of LA, BA, and SBP substrates were the most immune stimulatory, we compared particle size distribution of the suspensions (Figure 5). The particle size distributions of the three fibers were significantly different in terms of average median particle size and distribution pattern (Figure 5). The LA sample containing soluble and particulate material is normally distributed with one main population (80 volume %) in the range of 13–227 μm, with an average median LA particle size of 59 μm.

The particle size distribution of BA consisted of one main, normally distributed population and two smaller populations. The main population (80 volume %) ranges from 0.8 to 6.3 μm with a average size of 2.1 μm. The two other populations (together 20 volume %) have average sizes of 24.7 and 106.9 μm. The particle size distribution of suspended SBP consisted of an average particle size of 214 μm (38–439μm).

Despite the difference in particulate size of the BA and LA substrates, sieving the dry material into two fractions comprising small and large particles did not lead to different cytokine responses when incubated with BMDCs (data not shown). In contrast to BA and LA, the large sieved fraction of SBP, induced significantly lower amounts of cytokine than the small fraction (data not shown). This might be due to a lower solubility and increased heterogeneity of the large SBP fraction as previously shown with β-glucans (44). Overall, these results indicated that different particle size distributions do not influence immune stimulatory activity of LA and BA.

### Hydrodynamic volume of the arabinans by HPSEC

As many studies report that the molecular mass of dietary fibers such as glucans, mannans, galactans and fructans influences their immune stimulatory activity (20) we fractionated the arabinans by HPSEC and tested their immune stimulatory activity. Although the soluble fraction was less immunomodulatory than the particulate fiber fraction, the molecular mass of the three substrates was analyzed for differences with HPSEC. BA and LA have very similar average molecular mass of 78 and 72 kDa, respectively. Soluble SBP consists of molecules of higher molecular mass of 213 kDa. The higher molecular mass of SBP in comparison to LA and BA was expected, as BA and LA are fragments of the SBP. As the molecular masses of BA and LA were rather similar, it is unlikely that this is the reason for their different immunomodulatory activities.

### Monosaccharide composition of the soluble and particulate arabinans

As particulate BA and LA differed in their immune stimulatory activity (Figure 4), their monosaccharide composition was investigated. The monosaccharide composition (Table 2) of the parental BA consists of 74 mol% arabinose, 13 mol% galactose and 9 mol% uronic acid with a total monosaccharide content of 67 w/w%. The parental LA consists of 60 mol% arabinose, 17 mol% galactose and 13 mol% uronic acid with a total monosaccharide content of 69 w/w%. SBP consists mainly of α-1,4 linked galacturonic acid forming a backbone (78 mol%), which is 53 % methylesterified and 17% acetylated (45). Side chains of galactose (12 mol%) and arabinose (6 mol%) are attached to the rhamnose at the rhamnogalacturonan backbone with an average length of ca. 9 residues.

**Table 2:** Monosaccharide composition of the three selected fibers and their particulate and soluble fractions.

	**(mol%)**							
**Sample**	**Rha**	**Ara**	**Xyl**	**Man**	**Gal**	**Glc**	**Uronic acid**	**Total (w/w%)**	**Yield (w/w%)**
SBP	2	6	0	0	12	2	78	73	n.a.
BA	1	74	0	1	13	3	9	67	n.a.
LA	3	60	0	0	17	6	13	69	n.a.
Particulate SBP	3	6	0	0	18	1	71	72	63
Soluble SBP	1	7	0	0	9	2	81	75	37
Particulate BA	2	69	0	1	12	4	11	32	2
Soluble BA	2	74	0	0	12	3	8	74	98
Particulate LA	1	73	0	0	12	5	9	79	19
Soluble LA	3	53	0	0	22	6	16	63	81

*n.a., not analyzed*.

The yield of the particulate BA and LA fractions is 2 and 19 w/w% of the original sample respectively, and both are immune stimulating. The carbohydrate content of particulate BA and LA is 32 and 79 w/w% respectively, resulting in only 0.6 and 15 w/w% of particulate carbohydrate present in the non-fractionated substrates. Linearization of BA has been previously shown to cause precipitation (46), so the small amount of particulate BA (2% w/w) might in fact be linear and immune stimulating as shown for LA.

### Enzymatic linearization of BA alters its immune-stimulatory activity

As the commercial LA was more immune-stimulatory than BA or SBP, we investigated whether enzymatically linearization of BA could increase its immune-stimulatory activity. Incubation of BA with arabinofuranosidase removes the arabinose side chains connected to the arabinose backbone (47), thereby increasing the linearity of the molecule. The time of incubation with arabinofuranosidase influenced the immune stimulatory activity of resulting BA. For example, 4 h incubation with arabinofuranosidase significantly decreased immune stimulatory activity on BMDCs and secretion of TNF, whereas this increased again after 24 and 48 h incubation (Figure 7). Incubation of BA with arabinofuranosidase significantly increased amounts of secreted IL-6 and MCP-1 compared to untreated sample although peak immune stimulating activity was after 4–7 h not 24 h. As the enzyme treated BA was heated to inactive enzyme and then freeze dried before resuspension and incubation with BMDCs, we confirmed that the immune stimulatory activity of LA and BA was not affected by the freeze-drying process itself (data not shown). Linearization of BA has been previously shown to cause precipitation (46), suggesting the increased amount of particulate fiber was responsible for the altered immune stimulatory activity.

**Figure 6:**
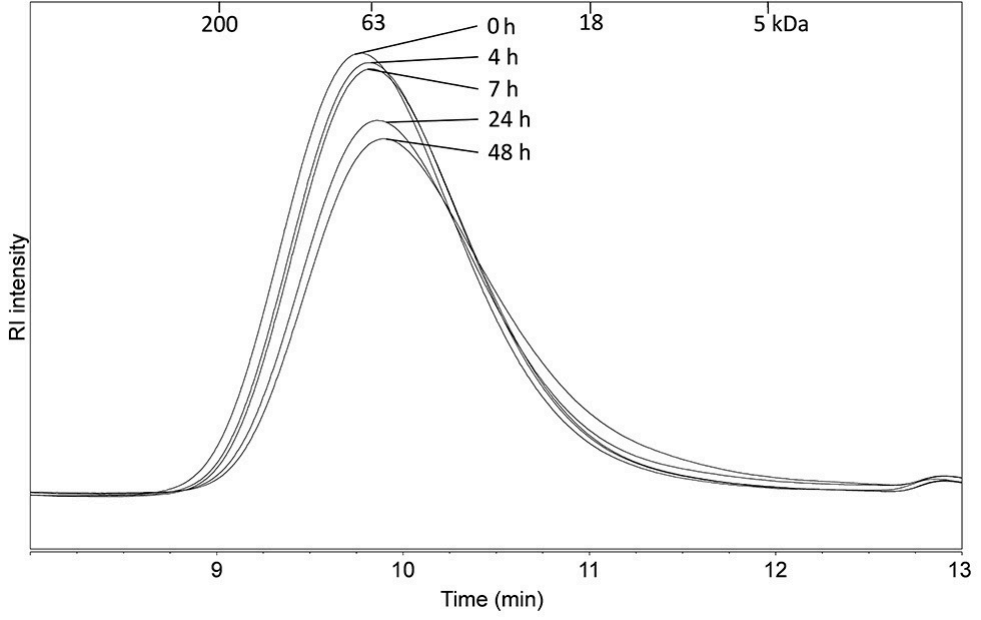
Molecular mass distribution of BA after 0, 4, 7, 24, and 48 h of incubation with α-arabinofuranosidase which removing the branched arabinoses but not causing a change in the molecular mass distribution.

Measurement of the molecular mass of the incubated BA samples showed that there was no shift to smaller molecular masses (Figure 6). This was expected, as the molecular mass is determined by the hydrodynamic volume of the longest backbone chain and the enzyme used is known to cleave off only the side chains (48). This finding suggests that the linearity of the arabinans but not the molecular mass, is influencing their immune stimulatory activities.

**Figure 7:**
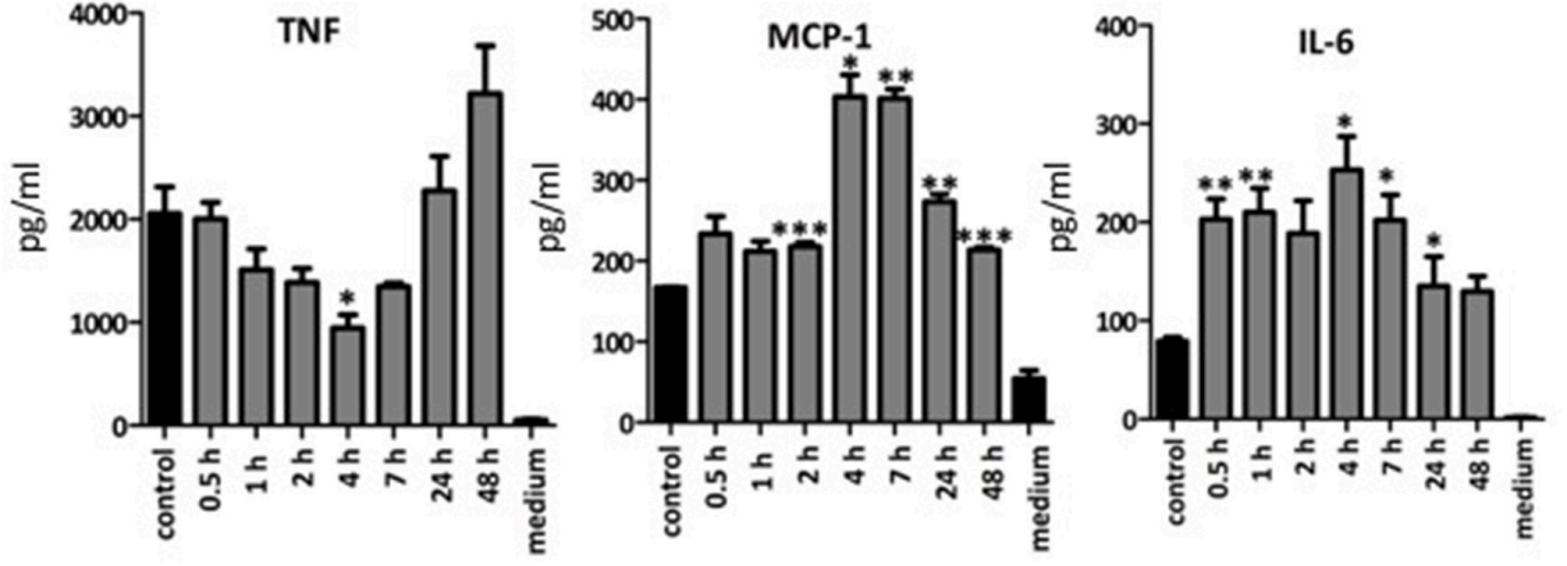
Mean amounts of cytokine or MCP-1 chemokine produced after incubation of BMDC from TLR2x4 double-KO mice with an enzymatically linearized suspension of BA. Error bars represent the standard error of the mean values. Asterisks represent classes of statistically significant different responses compared to the control (BA and enzyme, not incubated) (*P < 0.05, **P < 0.01, and ***P < 0.0001).

### The role of SYK in the cytokine response to BA and LA

Dectin-1 is one of several C-type lectin receptors possessing a single cytoplasmic hemi-ITAM motif that can recruit SYK kinase upon clustering of the receptor signaling complex. Even in MyD88 TRIF knockout mice, that lack TLR-dependent pathways of NF-κB activation, Dectin-1 binding to particulate β1,3-linked glucans can induce SYK-dependent activation of DCs and production of IL-10, IL-2, TNF-α, IL-23, and IL-6, but little IL-12 (49). To investigate whether the induction of IL-10, TNF, IL-12, and IL-6 were SYK-dependent we initially determined the dose-dependency of the cytokine response to SBP, BA and LA and the effect of piceatannol, a pharmacological inhibitor of SYK kinase, on cytokine secretion in BMDCs from TLR2x4 double-KO mice (50).

**Figure 8:**
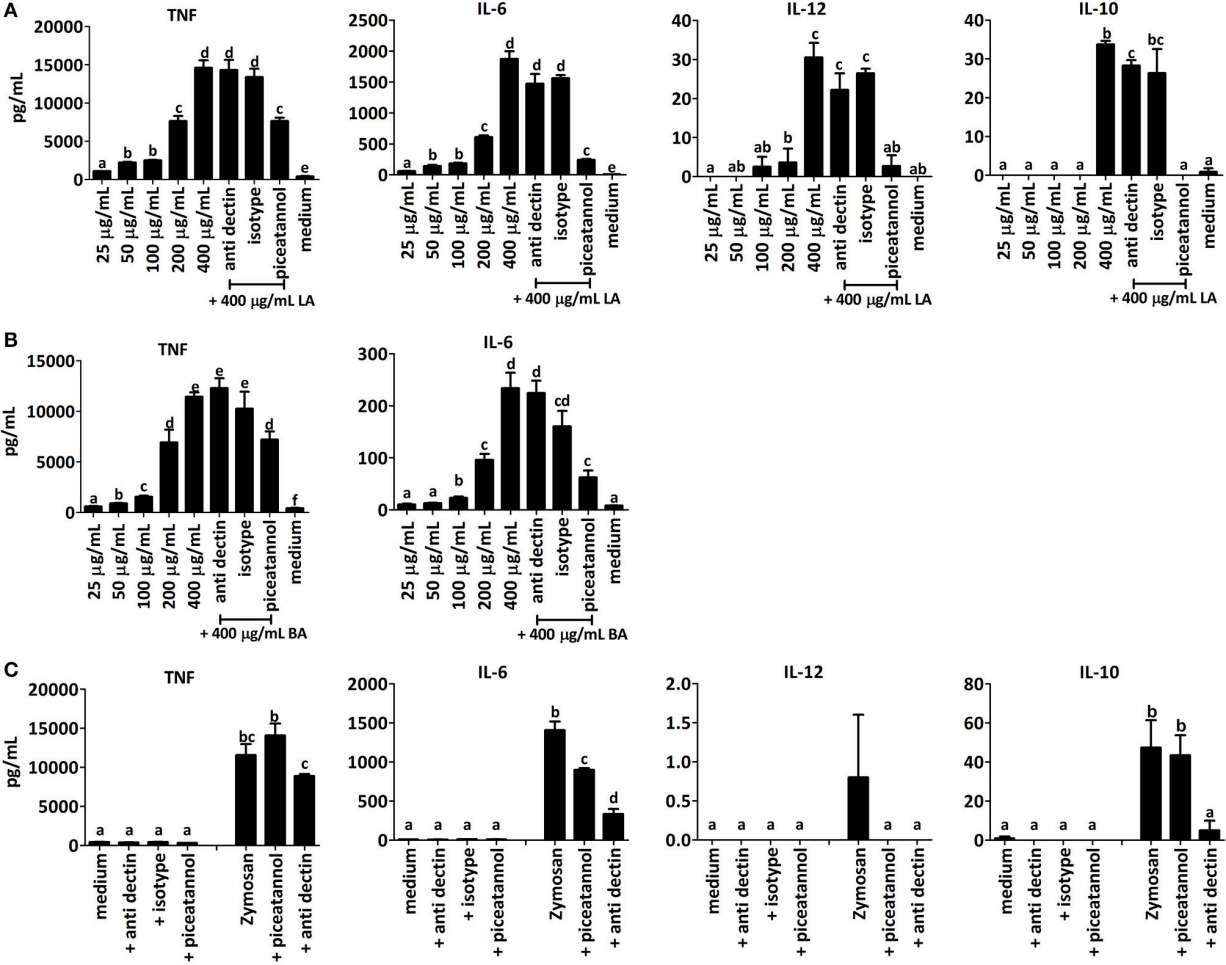
BMDC from TLR2x4 double-KO mice were incubated with different concentrations (25, 50, 200, and 400 μg/mL) of LA **(A)** or BA **(B)** and cytokine responses measured. Syk pathway inhibitor piceatannol (10 μM), Dectin-1 blocking antibody or an isotype control antibody were added to some samples LA or BA (400 μg/mL). In panel **(C)** BMDC from TLR2x4 double-KO mice were incubated with medium plus piceatannol (10 μM), anti-dectin-1 or an isotype antibody control. Similarly, cells were incubated with depleted zymosan in presence and absence of piceatannol (10 μM) or anti-dectin-1. Error bars represent the standard error of the mean values. Letters above the bar represent classes of statistically significant different responses compared to each stimulation; thus, in each graph samples with the same letter are not significantly different.

LA induced significant amounts of TNF, IL-6, IL-12, and IL-10 in BMDCs compared to the medium control, whereas at the same concentration, BA and SBP only induced TNF and IL-6 (Figure 8B). Furthermore, LA induced secretion of larger quantities of cytokines than BA and SBP in a concentration-dependent fashion (Figure 8). In BMDCs stimulated with LA at the highest dose (400 μg/mL), piceatannol significantly reduced the amount of secreted IL-6, IL-10, TNF, and IL-12 (Figure 8) suggesting that the fiber is inducing cytokines in a Syk-dependent manner. This was not due to an effect of the inhibitor on cell viability as shown by quantification of Annexin V and propidium iodide stained cells by flow cytometry (data not shown). A significant inhibitory effect of piceatannol on induction of cytokine secretion was also observed with BMDCs stimulated with BA and SBP. To investigate whether SBP, LA, and BA interacted with Dectin-1 receptor, we added a blocking antibody (anti Dectin blocking) to Dectin-1 prior to the incubation of the fibers with BMDCs and included an isotype control antibody in the assay to rule out non-specific effects of antibody binding. The anti-dectin antibody significantly reduced the IL-10 induction by LA, but had no significant effect on all other cytokines induced by LA or BA. To control for the ability of the anti-dectin antibody to block the Dectin-1 receptor we added it to BMDCs incubated with depleted zymosan, a known agonist of the Dectin-1 receptor. As expected anti-dectin strongly reduced the induction of IL-10 and IL-6 by depleted zymosan (Figure 8C). Taken together these results suggest that LA and BA activate BMDCs and induce cytokine secretion via a SYK-dependent pathway, and likely through a CLR other than Dectin-1.

## Discussion

There is much interest in the immune stimulatory effects of dietary polysaccharides, on immune cells, especially the fungal β-1,3 glucans which interact with the C-type lectin receptor Dectin-1 which plays a role in innate immunity to fungal pathogens. Nevertheless, the identification of dietary fibers with immunomodulatory effects is often confounded by contamination with microbe-associated molecular patterns such as LPS, which interact with TLRs to activate MyD88 and TRIF-dependent activation of innate immune responses. Effective removal or inactivation of all TLR agonists from plant fibers may be difficult without affecting physicochemical properties of the fiber and often LPS is the only MAMP measured reliably at the low concentrations triggering immune responses.

In an initial screen for the capacity of 44 fibers to activate NF-κB via different TLRs we found that many fibers activate NF-κB via TLR4, TLR2, TLR2/1, and TLR2/6 signaling, suggesting contamination with multiple MAMPs including LPS, acylated lipoproteins and lipoteichoic acids. There was no significant activation of NF-κB via TLR5 signaling which recognized flagellin a structural protein of the flagellum found in *Enterobacteriaceae* (not shown). In light of the significant presence of TLR signaling activity by some fibers we screened for immune stimulatory activity on BMDCs from mice lacking TLR2 and TLR4, even though it would not detect fibers which might interact with TLR2 and TLR4 as reported in some studies (37, 38). This approach was validated by incubation of BMDCs from TLR2x4 double-KO mice with a range of doses of different purified TLR agonists and measured secretion of several cytokines and the chemokine MCP-1 after 24 h. None of the TLR2 and 4 agonists tested elicited significant cytokine or chemokine secretion in BMDCs from KO mice while they induced strong immune responses in BMDCs from wild-type mice (data not shown). Surprisingly flagellin did not elicit cytokine responses in BMDCs from knockout or wild type mice, but a similar finding was previously described for flagellin (51). Purified zymosan lacking any TLR signaling activity induced immune responses in BMDCs from KO mice showing that the Dectin-1 signaling pathway was active and these cells could be used to measure TLR2 and 4 independent immune responses, elicited by CLRs of the innate immune system.

As three related fibers (SBP, LA, and BA) differed considerably in their capacity to elicit secreted cytokines in BMDCs we investigated how their physicochemical properties affect immune stimulatory activity. SBP and BA are both derived from sugar beet pulp by either acid or alkaline extraction (39), whereas LA is derived from BA by enzymatic treatment with an arabinofuranosidase (40). All three fibers LA, BA, and SBP induced cytokines in BMDCs from TLR2x4 double-KO mice, but LA appeared to be the most immune stimulatory. Separation of the fiber suspensions into soluble and particulate fractions by centrifugation clearly showed that most immune stimulatory activity was present in the re-suspended particulate fractions (Figure 3). This suggest that the arabinans must be presented in an immobilized form e.g., on the surface of a particle to induce robust signaling. This scenario is reminiscent of reports showing that particulate preparations of fungal β-1,3 glucans are required for signaling via the C-type lectin receptor Dectin-1. In the case of β-1,3 glucans, the binding of immobilized ligands clusters the Dectin-1 receptor, bridging the phosphorylated hemi-ITAM motifs present on the intracellular domain of this receptor, thereby allowing SYK to be recruited and trigger MyD88 and TRIF independent activation of NF-κB (44). A similar clustering of C-type lectin receptors binding to the arabinans may explain why the particulate fractions of BA and LA are more immune stimulatory than the soluble fractions.

Physical properties of BA and LA, such as molecular mass and particle size distribution did not strongly influence their individual immune stimulatory activity, suggesting chemical differences between LA and BA account for the stronger immune activation observed with LA. The chemical difference between LA and BA is in the degree of branching on the arabinan backbone, reflected in the higher amounts of arabinose in BA than in LA [Table 1, (46)]. As anticipated extensive enzymatic debranching of BA increased its immune stimulatory activity more than 3-fold. Previously, linearization of BA was shown to increase the amount of particulate fiber possibly due to the alignment of debranched linear molecules (46), which could explain its increased immune stimulatory activity.

To explore the possible involvement of SYK kinase in cytokine induction by LA and BA, we performed BMDC stimulation assays in the presence of piceatannol an inhibitor of SYK kinase that has also been reported to block β1,3 glucan-mediated activation of NF-κB (39). As shown in other experiments LA induced TNF, IL-10, IL-12, and IL-6 secretion in BMDCs from TLR2x4 double-KO mice, whereas BA induced only TNF and IL-6 (Figure 8). Piceatannol significantly inhibited the amounts of the different cytokines elicited by LA, although this effect was strongest for IL-10, IL-6, and IL-12. Similarly, piceatannol significantly inhibited IL-6 and TNF secretion elicited by BA.

Inclusion of a Dectin-1 blocking antibody or isotype control antibody to BMDCs stimulated with LA and BA did not significantly affect cytokine responses whereas the Dectin-1 blocking antibody did significantly reduced cytokine secretion in response to β1,3 glucan. Together, these results demonstrate that particulate arabinans derived from sugar beet pectin, best reflected by LA, are immune stimulatory, through a mechanism involving a SYK-dependent kinase.

Particulate arabinans present in food and digesta might encounter dendritic cells or macrophages associated with the epithelium or via uptake into Peyer's patches and modulate immune responses in the intestine. It is also hypothesized that the receptor mediating these responses to arabinans is expressed in cells of the epithelial lineage as reported for Dectin-1, which would have implications for direct innate signaling effects on the intestinal epithelium (52).

## Ethics statement

Specific pathogen free TLR2^−/−^ TLR4^−/−^ C57bl/6 mice, were housed in groups (*n* = 3) and received a standard chow diet and sterilized drinking water *ad libitum* in filter top cages. Euthanized mice were used to obtain bone marrow cells from the femur. The breeding and euthanasia of these mice was approved by the animal welfare committee of the Wageningen University (Wageningen, The Netherlands).

## Author contributions

MM, JW, CR, KV, and HS designed the experiments and these were performed by MM, NT, and CR. JW and MM wrote the first draft of the manuscript. MM, CR, NT, KV, HG, HS and JW contributed to the interpretation of the data and commented on the manuscript.

### Conflict of interest statement

The authors declare that the research was conducted in the absence of any commercial or financial relationships that could be construed as a potential conflict of interest.
