# Supplementary material for: Structure Dependent-Immunomodulation by Sugar Beet Arabinans via a SYK Tyrosine Kinase-Dependent Signaling Pathway
Source: Front Immunol. 2018 Oct 12;9:1972. doi: 10.3389/fimmu.2018.01972 (PMC6194903; doi:10.3389/fimmu.2018.01972)
Supplement: Supplementary file 1 [file Data_Sheet_1.PDF]

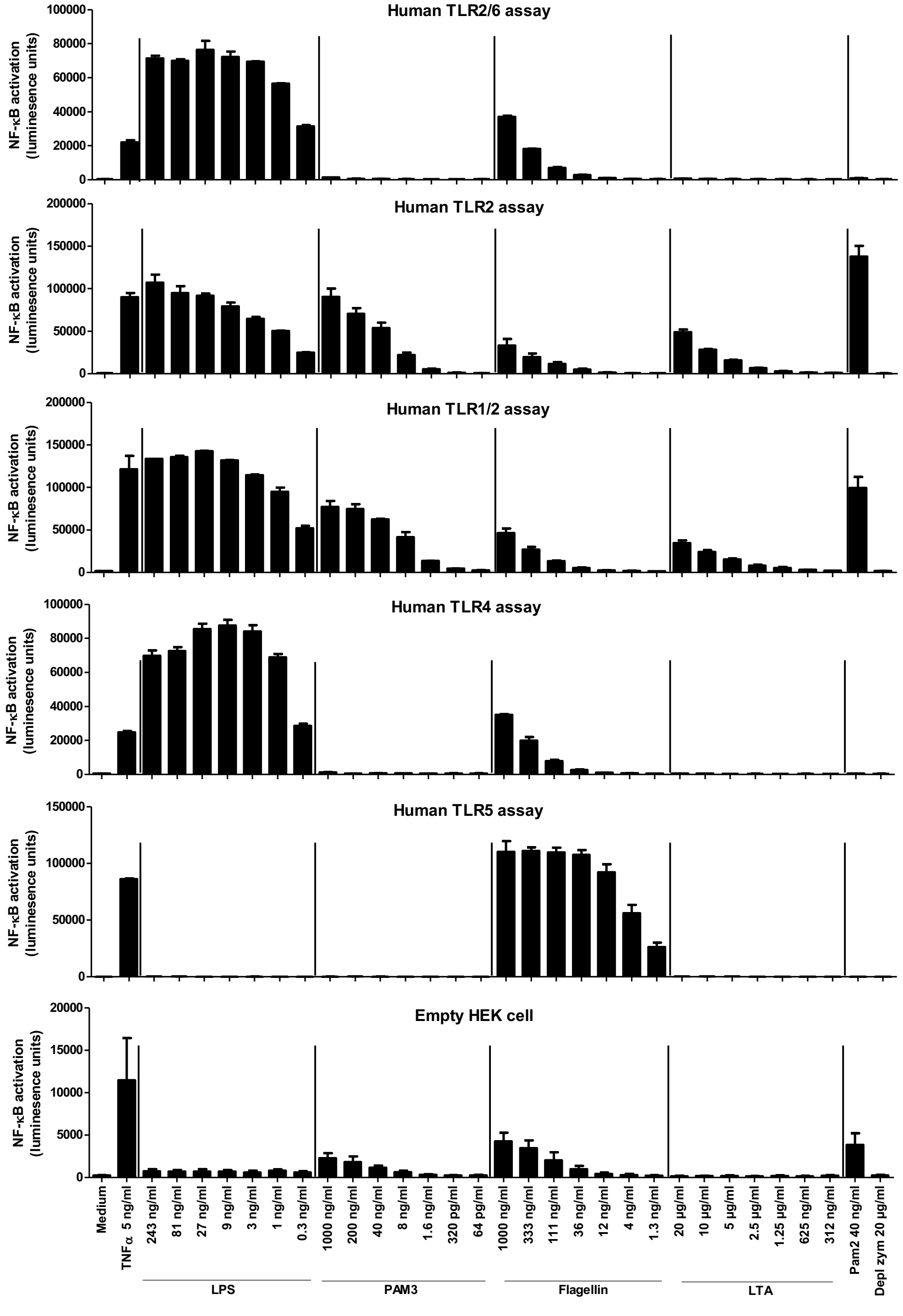

Supplementary figure 1: TLR signalling response to different ligands. HEK293 cells were incubated with LPS (1 ng/mL-750ng/mL), PAM3 (64 ng/mL-1 µg/mL), Flagellin (1.3 ng/mL-1 µg/mL), LTA (312 ng/mL-20µg/mL), PAM2 (40 ng/mL) and depleted Zymosan (20 µg/mL).
